# Polyphenolic Content, Antioxidant and Antimicrobial Activities of *Lycium barbarum* L. and *Lycium chinense* Mill. Leaves

**DOI:** 10.3390/molecules190710056

**Published:** 2014-07-10

**Authors:** Andrei Mocan, Laurian Vlase, Dan Cristian Vodnar, Cristina Bischin, Daniela Hanganu, Ana-Maria Gheldiu, Radu Oprean, Radu Silaghi-Dumitrescu, Gianina Crișan

**Affiliations:** 1 Department of Pharmaceutical Botany, Iuliu Hațieganu University of Medicine and Pharmacy, 12 I. Creangă Street, Cluj-Napoca 400010, Romania; E-Mails: amocanm@gmail.com (A.M.); gcrisan@umfcluj.ro (G.C.); 2 Department of Pharmaceutical Technology and Biopharmaceutics, Iuliu Hațieganu University of Medicine and Pharmacy, 12 I. Creangă Street, Cluj-Napoca 400010, Romania; E-Mail: anamaria.gheldiu@yahoo.com; 3 Department of Food Science and Technology, University of Agricultural Sciences and Veterinary Medicine, 3-5 Manăştur Street, Cluj-Napoca 400372, Romania; E-Mail: vodnar_dan@yahoo.co.uk; 4 Department of Chemistry and Chemical Engineering Babeș-Bolyai University, 11 A. Janos Street, Cluj-Napoca 400028, Romania; E-Mails: cristina_bischin@yahoo.com (C.B.); rsilaghi@chem.ubbcluj.ro (R.S.-D.); 5 Department of Pharmacognosy, Iuliu Hațieganu University of Medicine and Pharmacy, 12 I. Creangă Street, Cluj-Napoca 400010, Romania; E-Mail: handa_1964@yahoo.com; 6 Department of Analytical Chemistry and Instrumental Analysis, Iuliu Hațieganu University of Medicine and Pharmacy, 4 L. Pasteur Street, Cluj-Napoca 400010, Romania; E-Mail: roprean@umfcluj.ro

**Keywords:** *Lycium barbarum*, *Lycium chinense*, polyphenols, antioxidant compounds, antimicrobial activity

## Abstract

This study was performed to evaluate the *in vitro* antioxidant and antimicrobial activities and the polyphenolic content of *Lycium barbarum* L. and *L. chinense* Mill. leaves. The different leave extracts contain important amounts of flavonoids (43.73 ± 1.43 and 61.65 ± 0.95 mg/g, respectively) and showed relevant antioxidant activity, as witnessed by the quoted methods. Qualitative and quantitative analyses of target phenolic compounds were achieved using a HPLC-UV-MS method. Rutin was the dominant flavonoid in both analysed species, the highest amount being registered for *L. chinense*. An important amount of chlorogenic acid was determined in *L. chinense* and *L. barbarum* extracts, being more than twice as high in *L. chinense* than in *L. barbarum*. Gentisic and caffeic acids were identified only in *L. barbarum*, whereas kaempferol was only detected in *L. chinense.* The antioxidant activity was evaluated by DPPH, TEAC, hemoglobin ascorbate peroxidase activity inhibition (HAPX) and inhibition of lipid peroxidation catalyzed by cytochrome *c* assays revealing a better antioxidant activity for the *L. chinense* extract. Results obtained in the antimicrobial tests revealed that *L. chinense* extract was more active than *L. barbarum* against both Gram-positive and Gram-negative bacterial strains. The results suggest that these species are valuable sources of flavonoids with relevant antioxidant and antimicrobial activities.

## 1. Introduction

Recent important epidemiological studies have concluded that certain natural foods and medicinal plants can be involved in preventing or hindering the development of different diseases [1,2,3,4]. Interest in developing natural nutritional antioxidants is increasing due to their well documented impact on human health [5,6,7,8], but also to the fact that synthetic antioxidants have been incriminated as endocrine disrupters or even carcinogenic agents [1,2,9,10]. Considered to be the most frequent antioxidant compounds in human diets, polyphenols possess multiple biological properties [2,4,5,11], making it vital to learn about their amounts and varieties in medicinal plants and natural foods [12].

The importance of plants belonging to the genus *Lycium* L. (*Solanaceae*) has increased rapidly in the last few years due to their traditional usages in Chinese herbal medicine, and they are considered by most researchers as functional foods with a large variety of beneficial effects [13,14]. The *Lycium* genus comprises approximately 70 species which vegetate in separate and distinct regions distributed from the temperate to the subtropical regions of Eurasia, North America, South America, southern Africa and Australia [15,16]. The Romanian flora includes only two representatives of the *Lycium* genus, *Lycium barbarum* L. and *Lycium chinense* Mill. and mentions them as cultivated or sub-spontaneous species [17]. 

*L. barbarum* (Chinese wolfberry, Barbary wolfberry or Chinese boxthorn) has become more popular in the last few years due to its public acceptance as a superfood with highly advantageous nutritive and antioxidant properties [16,18]. Use of its fruits as a functional food are mentioned as far back as 2,800 B.C. in Chinese traditional medicine, but also the leaves are described as being consumed in tea infusions or as spices [16,19,20]. Among the chemical constituents described for *L. barbarum* fruit, the most well researched components are the water-soluble polysaccharides, estimated to comprise 5%–8% of the dried fruits [16]. Another compound class is the carotenoids group, mostly represented by zeaxanthin and its esters, which make up only 0.03%–0.5% of the dried fruit [16,21]. Generally, researchers from the Chinese scientific space have devoted their attention to polysaccharides [14,16,22,23], while those who are outside of China have studied other functional constituents including antioxidants, alkaloids, glycopeptides, glycoprotein and tocopherols [14]. Among other analyzed compounds in the *L. barbarum* fruit, the literature mentions small amounts of flavonoids, phenolic acids, sterols and betaine [12,16,20,24,25]. Recent studies indicate that extracts of *L. barbarum* fruits and one of its active components, the polysaccharides possess a large range of biological activities, including effects on aging [26], neuroprotection [27,28], anti-fatigue/endurance [29], hypoglycemic [30], increasing metabolism [22,31,32], glaucoma [28], anti-cancer activity and cytoprotection [33,34,35], immunomodulation [36,37,38,39], and antioxidant properties [40,41,42,43].

*L. chinense* Mill. or Chinese desert thorn is less known than *L. barbarum*, but also a traditional Chinese herb considered an ingredient for eternal youth and long life, a tonic that reduces the risk of arteriosclerosis and arterial hypertension [44]. Its fruits have drawn the attention of scientists due to their compounds, such as betaine, cerebrosides, glycolipds, polysaccharides which exhibit several important biological effects like hepatoprotection [45] and antioxidant [46,47]. The root barks present anti-inflammatory effects [48]. Leaves of *L. chinense* are used in tea infusions in the Orient, and nowdays are considered as a healthful food [44]. We could identify only limited publications that deal with the leave composition of these two *Lycium* species [20,34,44,49,50]. To increase our understanding of the pharmacological and nutraceutical properties of *L. barbarum* and *L. chinense* we employed a rapid, highly accurate and sensitive HPLC method assisted by MS detection for the simultaneous determination of 19 polyphenols [51,52]. Due to the fact that the chemical composition of Romanian cultivated *L. barbarum* and *L. chinense* has never the subject of a scientific paper to our knowledge, the aim of this work was to characterize the polyphenolic composition of leaves from *L. barbarum* and *L. chinense* and to evaluate their *in vitro* antioxidant and antibacterial activities using several assays.

## 2. Results and Discussion

### 2.1. HPLC Analysis of Polyphenols

**Figure 1:**
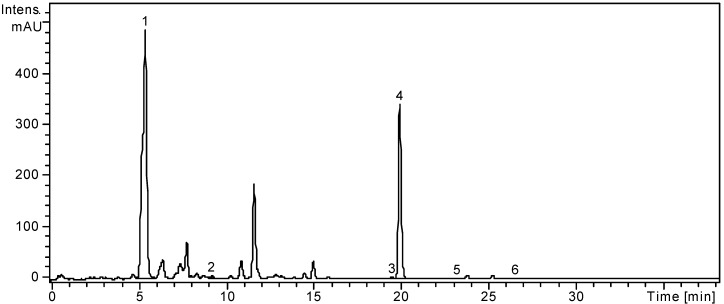
HPLC chromatogram of *L. barbarum* sample.

*Notes*: Chromatographic conditions as given in the Experimental Section. Identified compounds: 1, Chlorogenic acid; 2, *p*-Coumaric acid; 3, Isoquercitrin; 4, Rutin; 5, Quercitrin; 6, Quercetin.

**Figure 2:**
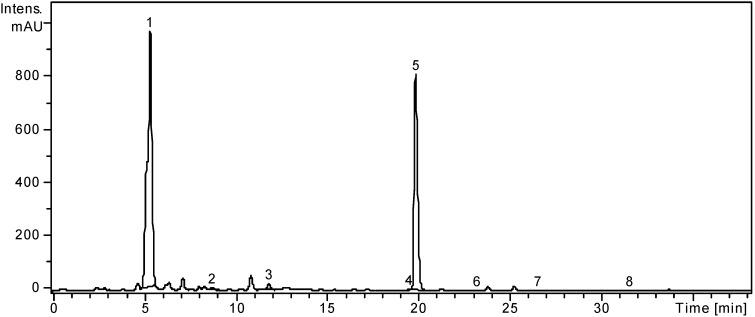
HPLC chromatogram of *L. chinense* sample.

*Notes*: Chromatographic conditions as given in the Experimental Section. Identified compounds: 1, Chlorogenic acid; 2, *p*-Coumaric acid; 3, Ferulic acid; 4, Isoquercitrin; 5, Rutin; 6, Quercitrin; 7, Quercetin; 8, Kaempferol.

The quantitative determination was performed using the external standard method. The concentrations of identified polyphenolic compounds were organized in order of their retention times and are presented in Table 1. The HPLC chromatograms of *L. barbarum* and *L. chinense* are presented in Figure 1 and Figure 2.

**Table 1:** The polyphenolic compounds content in the studied species (μg/g plant material).

Polyphenolic Compound	*m*/*z*	R_T_ ± SD (min)	*L. barbarum*	*L. chinense*
Gentisic acid	179	3.52 ± 0.04	<0.02	NF
Caffeic acid	179	5.60 ± 0.04	<0.02	NF
Chlorogenic acid	353	5.62 ± 0.05	5899.29 ± 4.46	12045.96 ± 9.25
*p*-Coumaric acid	163	9.48 ± 0.08	30.29 ± 0.23	54.97 ± 0.43
Ferulic acid	193	12.8 ± 0.10	<0.02	112.25 ± 0.87
Isoquercitrin	463	19.60 ± 0.10	25.08 ± 0.72	20.46 ± 0.21
Rutin	609	20.20 ± 0.15	5646.66 ± 3.32	16205.28 ± 8.09
Quercitrin	447	23.64 ± 0.13	13.00 ± 0.12	5.52 ± 0.07
Quercetin	301	26.80 ± 0.15	5.59 ± 0.06	4.49 ± 0.05
Kaempferol	285	32.48 ± 0.17	NF	2.83 ± 0.03

Note: NF—not found, below limit of detection. Values are the mean ± SD (*n* = 3).

Gentisic, caffeic, chlorogenic, *p*-coumaric, ferulic acids were identified in the ethanolic extract of *L. barbarum*, and chlorogenic and *p-*coumaric acids were quantified (5899.29 ± 4.46 μg/g, 30.29 ± 0.23 μg/g). Duan *et al.*, determined the amount of gentisic acid from *L. barbarum* leave methanolic extracts by using a capillary electrophoresis method [49]. Regarding the presence of caffeic, chlorogenic, *p*-coumaric and ferulic acids, this is the first report that mentions the presence of caffeic and ferulic acids and quantifies chrologenic and *p*-coumaric acids in *L. barbarum* leaves. Among the identified flavonoid glycosides, rutin is the main flavonoid in *L. barbarum* leaves (5646.66 ± 3.32 μg/g), as already reported by Dong *et al.* [20] and Duan *et al.* [49] and its amount is lower than those authors stated. One flavonoid aglycone, quercetin, could be quantified (5.59 ± 0.06 μg/g) and no previous data was found regarding its presence in *L. barbarum* leaves.

In the ethanolic extract of *L. chinense*, three hydroxicinnamic acid derivates, namely chlorogenic acid, *p*-coumaric acid and ferulic acid were identified and quantified (Table 1). The highest amount was determined for chlorogenic acid (12045.96 ± 9.25 μg/g). The presence of chlorogenic acid and rutin in the leaves of *L. chinense* was also mentioned by Terauchi *et al*. in 1997 and quantified by Qian *et al.* in 2004 and their amounts are comparable with the results obtained by Qian *et al.* [14,50]. *p*-Coumaric and ferulic acids were not mentioned before for *L*. *chinense* leaves. Protocatechuic acid was already determined by Qian *et al.* in aqueous and ethanolic extracts of *L. chinense* leaves. Three flavonoid glycosides, isoquercitrin (quercetin 3-glucoside), rutin (quercetin-3-*O*-rutinoside) and quercitrin (quercetin 3-rhamnoside) could be identified and quantified as seen in Table 1, with rutin being the predominant flavonoid (16205.28 ± 8.09 μg/g). Chinese authors also mention rutin as the dominant flavonoid but no previous data were found regarding the presence of isoquercitrin and quercitrin. Other flavonoids like hesperidin were identified and hyperoside, morin and quercetin were quantified by the same Chinese authors in *L. chinense* leaves ethanolic extracts [14]. Free flavonoid aglycones, quercetin and kaempferol were found in small quantities (4.49 ± 0.05, and 2.83 ± 0.03 μg/g, respectively). The presence of quercetin in small amounts was also signaled by Qian *et al.* [14], but no previous information was found regarding the free aglycone, kaempferol in *L. chinense* leaves.

Considering the 19 standard compounds used in this study, some other peaks were not identified. The comparative study showed significant differences in the composition of the investigated species, especially quantitative ones, regarding the amounts of rutin and chlorogenic acid as polyphenols. A one-way ANOVA test applied on the concentrations values of the identified compounds listed in Table 1 showed that there is a highly significant difference between these two extracts (*p* < 0.001).

### 2.2. Determination of Phenolic Compounds Content

The results of the amount of total polyphenolic contents (TPC), flavonoids and caffeic acid derivatives in the two analyzed species are represented in Table 2. Thus, the TPC values were expressed as gallic acid equivalents (mg GAE/g plant material). The calculation of total flavonoid content was carried out using the standard curve of rutin and presented as rutin equivalents (mg RE/g plant material) and the phenolic acids contents were expressed as caffeic acid equivalents (mg CAE/g plant material).

**Table 2:** The content of total polyphenols, flavonoids and caffeic acid derivatives in the extracts.

Samples	TPC (mg GAE/g Plant Material)	Flavonoids (mg RE/g Plant Material)	Caffeic Acid Derivatives (mg CAE/g Plant Material)
*L. barbarum*	61.59 ± 1.68	43.73 ± 1.43	16.95 ± 0.57
*L. chinense*	80.64 ± 2.02	61.65 ± 0.95	18.80 ± 0.61

Each value is the mean ± SD of three independent measurements. TPC: Total polyphenols content; GAE: Gallic acid equivalents; RE: rutin equivalents; CAE: caffeic acid equivalents.

The extract of *L. chinense* contained the highest amount of polyphenols, flavonoidic compounds and caffeic acid derivatives (80.64 ± 2.02, 61.65 ± 0.95, and 18.80 ± 0.61 mg/g respectively). Lower quantities were measured for the *L. barbarum* extract (61.59 ± 1.68, 43.73 ± 1.43, and 16.95 ± 0.57 mg/g respectively). As we can already notice, the flavonoids are the major polyphenolic compounds for both species. Comparing the result for *L. barbarum* samples with Dong *et al.* we can conclude that our samples were richer in flavonoids than what Chinese authors reported, but the amount of rutin, as main flavonoidic compound was lower [20]. No previous data regarding the total amounts of polyphenols, flavonoids and caffeic acid derivatives in *L. chinense* was found. The obtained results for this study suggest that both species can be considered as important source of flavonoids.

### 2.3. Antioxidant Activity

**Figure 3:**
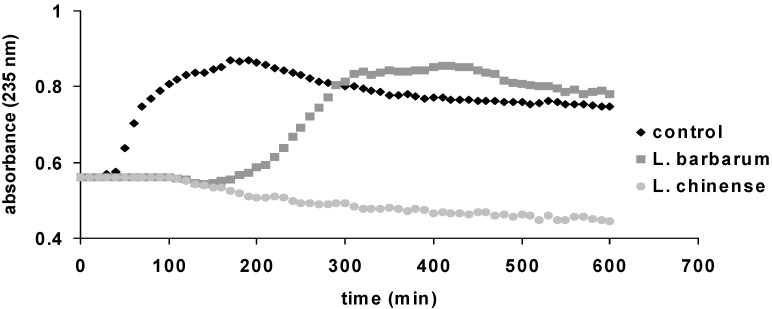
Liposome oxidation by cytochrome *c*, in the presence of the tested samples.

Note: Monitoring at 235 nm (specific for lipid oxidation).

The antioxidant activity of the ethanolic extracts of *L. barbarum* and *L. chinense* leaves was evaluated using the DPPH bleaching assay, Trolox equivalent antioxidant capacity (TEAC) method, hemoglobin ascorbate peroxidase activity inhibition (HAPX) assay and by testing the inhibition of lipid peroxidation catalyzed by cytochrome *c*, as shown in Table 3 and Figure 3.

**Table 3:** Antioxidant capacity parameters obtained using several methods for studied samples.

Samples	DPPH (µg QE/mg Plant Material)	TEAC (µg TE/mg Plant Material)	HAPX (%)
*L. barbarum*	29.30 ± 4.34	35.72 ± 6.29	29.69 ± 2.21
*L. chinense*	36.80 ± 0.65	55.95 ± 0.88	40.86 ± 2.21

Each value is the mean ± SD of three independent measurements. QE: Quercetin equivalents; TE: Trolox equivalents.

The antioxidant activity of the two ethanol extracts was assessed by the DPPH radical bleaching method and the results were presented as quercetin equivalents (Table 3). The highest radical scavenging activity was shown by *L. chinense* (36.80 ± 0.65 µg QE/mg plant material), while the *L. barbarum* extract exhibited a lower, but also important antioxidant activity. In this case, the percentage of DPPH consumption was converted to quercitin equivalents by using a calibration curve (*R*^2^ = 0.99) with quercetin standard solutions of 0–12 µM. The higher the rate of DPPH consumption is, the more powerful the antioxidant capacity.

The TEAC results are in agreement with the DPPH values and are also correlated with HAPX results and with total polyphenols, flavonoids and caffeic acid derivatives. DPPH and TEAC assays are both based on the same principle (free radical scavenging by electron transfer mechanism) and use synthetic radicals which react directly with antioxidants to quantify the antioxidant capacity of the sample; the notable difference is that in case of TEAC and HAPX assays, the solution is aqueous rather than ethanolic.

The newly developed and more physiologically relevant enzymatic assay (HAPX method) measures the capability of the extract components to quench the damage inflicted by hydrogen peroxide upon hemoglobin. This contributes with additional valuable information since it implies the interaction of the antioxidants molecules with a protein, *i.e.*, the physiological-relevant ferryl hemoglobin species (resulted by the action of hydrogen peroxide upon ferric hemoglobin) [53,54].

Another complex and arguably more physiologically relevant method based on peroxidase activity of cytochrome *c* was developed recently to evaluate the antioxidant capacity of the two ethanolic extracts. This process monitors the formation of lipid conjugated dienes at 235 nm. The antioxidant capacity of the tested extracts, reflected in the delay of the onset of lipid oxidation, is expected to be based on the same mechanism found in HAPX: the interaction of antioxidants with ferryl, generated in this case in cytochrome *c* [54,55]. In the lipid oxidation experiments, both extracts also demonstrated an antioxidant capacity and a good correlation with the TEAC and DPPH results, by inhibition of lipid peroxidation catalyzed by cytochrome *c* (Figure 3). The *L. barbarum* extract delays the oxidation of lipids about 100 min, while the *L. chinense* extract completely blocks the oxidation during the time of the experiment (600 min). According to Yang *et al.* the inhibition of lipid peroxidation is in direct correlation with increasing concentrations of rutin [56]. Thus *L. chinense* extract possesses a higher antioxidant activity.

The antioxidant activities of *L. barbarum* and *L. chinense* extracts were explored using four different tests, the simplest and traditionally TEAC and DPPH assays and two more complex and physiologically new relevant methods based on peroxidase activity of hemoglobin and cytocrome *c*. The antioxidant activity of vegetal extracts is strongly related with their chemical composition. As a peculiarity, *L. chinense* and *L. barbarum* leaves contain important amounts of flavonoids, from which the major compound was rutin and chlorogenic acid. High concentrations of rutin and chlorogenic acid are reflected in significant scavenging properties [56,57]. Comparing with our extracts, same concentrations of standard rutin exhibit lower antioxidant properties [56]. This is in line with the work of Terauchi *et al.* and Qian *et al.* and sustain that the antioxidant potential of these extracts is correlated with the amounts of rutin and chlorogenic acid and also influenced by the presence of other compounds as seen in Table 1 [14,50]. Comparing with other medicinal representatives that subjected the same antioxidant assays, *L. chinense* exhibits higher antioxidant activity than *Achillea distans* subsp. *alpina* and *Ocimum basilicum* [51,52].

In conclusion, results show a good correlation between methods as well as with the content of the total polyphenols, flavonoids and caffeic acid derivatives, with a notable antioxidant activity in both extracts. The highest activity is seen for *L. chinense*. There was no significantly statistical difference between the analyzed extracts in the DPPH assay (*p* > 0.05), but significant differences in TEAC and HAPX methods (0.001 < *p* < 0.05).

### 2.4. Antimicrobial Activity

Plants are important source of potentially useful structures for the development of new chemotherapeutic agents. The first step towards this goal is the *in vitro* antibacterial activity assay [58]. The results of testing the *L. barbarum* and *L. chinense* extracts for antimicrobial activities against both Gram-positive and Gram-negative bacteria are summarized in Table 4. Results obtained in the present study relieved that *L. chinense* extract was found to be more active than *L. barbarum* against both Gram-positive and Gram-negative bacterial strains. The best antibacterial activity was shown by *L. chinense* extract against *Bacillus subtilis*.

**Table 4:** Antibacterial activity of *L. barbarum* and *L. chinense* extracts and antibiotic against bacterial species tested by disc diffusion assay.

Bacterial Strains	Standard Antibiotic	Inhibition Zone (mm)	
	Gentamicin	*L. barbarum*	*L. chinense*
*Staphylococcus aureus*	9.1 ± 0.9	13.1 ± 0.9	12.1 ± 0.9
*Bacillus subtilis*	17.2 ± 0.8	17.2 ± 0.6	24.2 ± 0.6
*Listeria monocytogenes*	12.3 ± 0.8	13.1 ± 0.3	21.6 ± 0.8
*Escherichia coli*	12.3 ± 0.9	12.3 ± 0.8	14.5 ± 0.4
*Salmonella typhimurium*	15.1 ± 0.8	12.4 ± 0.7	19.6 ± 0.3

Each value is the mean ± SD of three independent measurements.

The strains of *L. monocytogenes* and *S. typhimurium* are also sensitive to the *L. chinense* extract with a zone of inhibition between 19–21 mm of diameter. The results obtained from the antimicrobial properties can make *L. chinense* a source of antibiotic having inhibited microbial growth. This is in line with the work of Dahech *et al.* [1].

The MIC values obtained from antimicrobial tests ranged from 50 to >100 µg/mL (Table 5). The results showed that the bacterial strains *S*. *typhimurium* was the most sensitive to both *L. barbarum* and *L. chinense* extracts with MIC value of 75 µg/mL and 50 µg/mL, respectively. Alternatively, *S. aureus* and *L. monocytogenes* were the least sensitive strains for both *Lycium sp.* extracts with MIC value >100 µg/mL. According to Salvat *et al.* plant extracts with MIC’s less than/or around 0.5 mg/mL indicate good antibacterial activity. Accordingly, *L. chinense* and *L. barbarum* extracts exhibited good antimicrobial activity against most of the tested microorganisms [59].

**Table 5:** Minimal Inhibitory Concentration (MIC) of both *L. barbarum* and *L. chinense* extracts.

Bacterial Strains	MIC (µg/mL)	
	*L. barbarum*	*L. chinense*
*Staphylococcus aureus*	>100	>100
*Bacillus subtilis*	100	75
*Listeria monocytogenes*	>100	>100
*Escherichia coli*	100	75
*Salmonella typhimurium*	75	50

Each value is the mean ± SD of three independent measurements.

## 3. Experimental Section

### 3.1. Plant Materials and Extraction Procedure

The vegetal material from *L. barbarum* (Voucher No. 3574) and *L. chinense* (Voucher No. 3575) species was purchased from local cultivators from Cluj-Napoca, Romania in the summer of 2013. Voucher specimens were deposited in the Department of Pharmaceutical Botany Herbarium of the Faculty of Pharmacy, “Iuliu Hatieganu” University of Medicine and Pharmacy, Cluj-Napoca, Romania. The leaves were air dried at room temperature in shade, separated and grinded to fine powder (300 µm). Twenty grams of each sample were weighed and extracted with 200 mL of of 70% ethanol for 30 min in a ultrasonication bath at 60 °C. The samples were then cooled down and centrifuged at 4,500 rpm for 15 min, and the supernatant was recovered. Stock standard solutions were prepared by accurately weighing 10 mg of chlorogenic, *p*-coumaric, caffeic, cichoric, caftaric, ferulic, sinapic, gentisic gallic acids, rutin, quercetin, isoquercitrin, quercitrin, hyperoside, kaempferol, myricetol, fisetin, patuletin, apigenin, luteolin, reference standards into separate 10 mL volumetric flasks and dissolving them in methanol [51,52].

### 3.2. Chemicals and Instrumentation

Chlorogenic acid, *p*-coumaric acid, caffeic acid, rutin, apigenin, quercetin, isoquercitrin, quercitrin, hyperoside, kaempferol, myricetol, fisetin from Sigma (St. Louis, MO, USA), ferulic acid, sinapic acid, gentisic acid, gallic acid, patuletin, luteolin from Roth (Karlsruhe, Germany), cichoric acid, caftaric acid from Dalton (Toronto, ON, Canada). HPLC grade methanol, ethanol, analytical grade orthophosphoric acid, hydrochloric acid and Folin-Ciocalteu reagent were purchased from Merck (Darmstadt, Germany), hydrogen peroxide, ABTS (2,2'-azinobis-3-ethylbenzotiazoline-6-sulphonic acid), sodium molybdate dihydrate, sodium nitrite, sodium hydroxide, sodium carbonate, sodium acetate trihydrate, and anhydrous aluminum chloride were from Sigma-Aldrich (Steinheim, Germany). DPPH (2,2-diphenyl-1-picrylhydrazyl) and Trolox (6-hydroxy-2,5,7,8-tetramethylchroman-2-carboxylic acid) were obtained from Alfa-Aesar (Karlsruhe, Germany), HRP (horseradish peroxidase) was purchased from Sigma-Aldrich. Bovine hemoglobin was purified following the general protocol of Antonini and Brunori [60]. The met forms of hemoglobin were prepared by ferricyanide treatment as previously described [61]. Liposomes were obtained by suspending 5 mg/mL soybean lecithin (Alfa Aesar) in phosphate buffer followed by sonication and horse heart purified cytochrome *c* from Sigma-Aldrich [54]. All spectrophotometric data were acquired using a Jasco V-530 UV-Vis spectrophotometer (Jasco International Co., Ltd., Tokyo, Japan).

### 3.3. HPLC-MS Analysis

#### 3.3.1. Apparatus and Chromatographic Conditions for the Analysis of Polyphenols

The identification and quantification of polyphenolic compounds was carried out using an Agilent Technologies 1100 HPLC Series system (Agilent, Santa Clara, CA, USA) Equipped with G1322A degasser, G13311A binary gradient pump, column thermostat, G1313A autosampler and G1316A UV detector. The HPLC system was coupled with an Agilent 1100 mass spectrometer (LC/MSD Ion Trap SL). For the separation, a reverse-phase analytical column was employed (Zorbax SB-C18 100 × 3.0 mm i.d., 3.5 μm particle); the work temperature was 48 °C. The detection of the compounds was performed on both UV and MS mode. The UV detector was set at 330 nm until 17.5 min, then at 370 nm. The MS system operated using an electrospray ion source in negative mode. The chromatographic data were processed using ChemStation and DataAnalysis software from Agilent. The mobile phase was a binary gradient: methanol and acetic acid 0.1% (v/v). The elution started with a linear gradient, beginning with 5% methanol and ending at 42% methanol, for 35 min; then 42% methanol for the next 3 min [2,51,52]. The flow rate was 1 mL·min^−1^ and the injection volume was 5 µL.

The MS signal was used only for qualitative analysis based on specific mass spectra of each polyphenol. The MS spectra obtained from a standard solution of polyphenols were integrated in a mass spectra library. Later, the MS traces/spectra of the analyzed samples were compared to spectra from library, which allows positive identification of compounds, based on spectral match. The UV trace was used for quantification of identified compounds from MS detection. Using the chromatographic conditions described above, the polyphenols eluted in less than 40 min (Table 6). Four polyphenols cannot be quantified in current chromatographic conditions due overlapping (caftaric acid with gentisic acid and caffeic acid with chlorogenic acid). However, all four compounds can be selectively identified in MS detection (qualitative analysis) based on differences between their pseudo-molecular mass and MS spectra. For all compounds, the limit of quantification was 0.5 μg/mL, and the limit of detection was 0.1 μg/mL. The detection limits were calculated as minimal concentration producing a reproductive peak with a signal-to-noise ratio greater than three. Quantitative determinations were performed using an external standard method. Calibration curves in the 0.5–50 μg/mL range with good linearity (*R*^2^ > 0.999) for a five point plot were used to determine the concentration of polyphenols in plant samples [2,51,52].

**Table 6:** Retention times (R_T_) of polyphenolic compounds (min).

Peak No.	Phenolic Compound	*m*/*z*	R_T_ ± SD	Peak No.	Phenolic Compound	*m*/*z*	RT ± SD
1.	Caftaric acid	311	3.54 ± 0.05	11.	Rutin	609	20.76 ± 0.15
2.	Gentisic acid	153	3.69 ± 0.04	12.	Myricetin	317	21.13 ± 0.12
3.	Caffeic acid	179	6.52 ± 0.04	13.	Fisetin	285	22.91 ± 0.15
4.	Chlorogenic acid	353	6.43 ± 0.05	14.	Quercitrin	447	23.64 ± 0.13
5.	*p*-Coumaric acid	163	9.48 ± 0.08	15.	Quercetin	301	27.55 ± 0.15
6.	Ferulic acid	193	12.8 ± 0.10	16.	Patuletin	331	29.41 ± 0.12
7.	Sinapic acid	223	15.00 ± 0.10	17.	Luteolin	285	29.64 ± 0.19
8.	Cichoric acid	473	15.96 ± 0.13	18.	Kaempferol	285	32.48 ± 0.17
9.	Hyperoside	463	19.32 ± 0.12	19.	Apigenin	279	39.45 ± 0.15
10.	Isoquercitrin	463	20.29 ± 0.10				

Note: SD, standard deviation.

#### 3.3.2. Identification and Quantification of Polyphenols

The detection and quantification of polyphenols was performed in UV assisted by mass spectrometry detection. Due to peak overlapping, four polyphenol-carboxylic acids (caftaric, gentisic, caffeic, chlorogenic) were determined only based on MS spectra, whereas for the rest of the compounds the linearity of the calibration curves was very good (*R*^2^ > 0.998), with detection limits in the range of 18 to 92 ng/mL. The detection limits were calculated as the minimal concentration yielding a reproducible peak with a signal-to-noise ratio greater than three. Quantitative determinations were performed using an external standard method; retention times were determined with a standard deviation ranging from 0.04 to 0.19 min (Table 6). For all compounds, the accuracy was between 94.1.3% and 105.3%. Accuracy was checked by spiking samples with a solution containing each polyphenol in a 10 μg/mL concentration. In all analyzed samples the compounds were identified by comparison of their retention times and recorded electrospray mass spectra with those of standards in the same chromatographic conditions.

### 3.4. Determination of Total Polyphenols, Flavonoids Content and Caffeic Acid Derivatives

The total phenolic content (TPC) of the extracts was measured using the Folin-Ciocalteu method with some modifications [51,52]. 2 mL from each ethanolic extract were diluted 25 times and then mixed with Folin-Ciocalteu reagent (1 mL) and distilled water (10.0 mL) and diluted to 25.0 mL with a 290 g/L solution of sodium carbonate. The samples were incubated in the dark for 30 min. The absorbance was measured at 760 nm, using a JASCO UV-Vis spectrophotometer. Standard curve was prepared by using different concentrations of gallic acid and the absorbances were measured at 760 nm. TPC values were determined using an equation obtained from the calibration curve of gallic acid graph (*R*^2^ = 0.999). Total polyphenolic content was expressed as mg gallic acid/g dry material plant (mg GAE/g plant material).

The total flavonoids content was calculated and expressed as rutin equivalents after the method described in the Romanian Pharmacopoeia (Xth Edition) for *Cynarae folium* [62]. Each extract (5 mL) was mixed with sodium acetate (5.0 mL, 100 g/L), aluminum chloride (3.0 mL, 25 g/L), and made up to 25 mL in a calibrated flask with methanol. Each solution was compared with the same mixture without reagent. The absorbance was measured at 430 nm. The total flavonoids content values were determined using an equation obtained from calibration curve of the rutin graph (*R*^2^ = 0.999).

The total content of caffeic acid derivatives was determined by using the spectrophotometric method with Arnow’s reagent (10 g sodium nitrite and 10 g sodium molybdate made up to 100 mL with distilled water) [51,52]. The percentage of phenolic acids, expressed as caffeic acid equivalent on dry material plant (mg CAE/g plant material), was determined using an equation that was obtained from calibration curve of caffeic acid (*R*^2^ = 0.994). For all methods, each sample was analyzed in triplicate.

### 3.5. In Vitro Antioxidant Activity Assays

#### 3.5.1. DPPH Bleaching Assay

The DPPH assay provides an easy and rapid way to evaluate potential antioxidants. DPPH free radical method is an antioxidant assay based on electron-transfer that produces a violet solution in ethanol. This free radical, stable at room temperature is reduced in the presence of an antioxidant molecule, giving rise to a yellow solution. The free radical scavenging activity of the ethanolic extracts was measured in terms of hydrogen donating or radical scavenging ability using this method. A stock solution of 100 µM DPPH was prepared. In a glass cuvette, 2 µL from original extracts were added to 998 µL DPPH solution. The absorbance changes were monitored at 517 nm for 30 min, using a UV-vis spectrophotometer equipped with a multi-cell holder. The percentage of DPPH consumption in each case was converted to quercetin equivalents using a calibration curve (*R*^2^ = 0.991) with quercetin standard solutions of 0–12 µM [51,52]. The higher the rate of DPPH consumption is, the more powerful the antioxidant capacity.

#### 3.5.2. TEAC Assay (Trolox Equivalent Antioxidant Capacity)

In the Trolox equivalent antioxidant capacity (TEAC) assay, the antioxidant capacity is reflected in the ability of the natural extracts to decrease the color, reacting directly with the ABTS radical. The latter was obtained by oxidation of ABTS (2,2'-azinobis(3-ethylbenzothiazoline-6-sulfonic acid)) with peroxide, catalyzed by HRP (horseradish peroxidase). Original extracts were diluted 5 times, and 3 µL from the diluted extract were added to 997 µL ABTS solution. The amount of ABTS radical consumed by the tested compound was measured at 735 nm, after 30 min of reaction time. The evaluation of the antioxidant capacity was obtained using the total change in absorbance at this wavelength. The percentage of ABTS consumption was transformed in Trolox equivalents (TE) using a calibration curve (*R*^2^ = 0.986) with Trolox standard solutions of 0–16 µM [51,52].

#### 3.5.3. Hemoglobin/Ascorbate Peroxidase Activity Inhibition (HAPX) Assay

Inhibition of hemoglobin ascorbate peroxidase activity assay (HAPX) was conducted according to the procedure described by Mot *et al.* [53]. Hemoglobin was purified according to Antonini and Brunori protocols [60]. The reaction was triggered by the addition of met hemoglobin (6 µM) to a mixture of ascorbate (160 µM), peroxide (700 µM) and extracts (5 µM) from the stock diluted 5 times, and it was monitored at 405 nm. This method allows us to evaluate the inhibition of ferryl formation by ascorbate in the presence of the tested compounds. An increase in the time of inhibition reflects the antioxidant capacity of the compound, whereas a decrease, a prooxidant effect [61].

#### 3.5.4. Inhibition of Lipid Peroxidation Catalyzed by Cytochrome *c*

Liposomes were obtained by suspending 5 mg/mL soybean lecithin in phosphate buffer (20 mM, pH 7), followed by sonication for 15 min in an ultrasonic bath (using a Power Sonic 410 device). The liposome oxidation experiment was performed at room temperature, for 600 min, in the presence of cytochrome *c* (2 µM) and extracts (5 µL from the diluted extract) by monitoring the absorbance at 235 nm (wavelength specific for liposome oxidation). This process monitors the formation of lipid conjugated dienes at the specified wavelength [54].

### 3.6. Determination of Antimicrobial Activity

#### 3.6.1. Microorganisms and Culture Growth

The microorganisms used for antimicrobial activity evaluation were obtained from the University of Agricultural Sciences and Veterinary Medicine Cluj Napoca, Romania. The bacteria strains were chosen due to their pathogenicity and implications in human health. Some of them subjected previous studies on antimicrobial activity of *Lycium* genus representatives [1]. The Gram-positive bacteria tested were *Staphylococcus aureus* (ATCC-25923), *Bacillus subtilis* (ATCC-12228), *Listeria monocytogenes* (ATCC-19115) and the Gram-negative ones *Escherichia coli* (ATCC-25922) and *Salmonella typhimurium* (ATCC-14028). The stock cultures of microorganisms used in this study were maintained on plate count agar slants at 4 °C. Inoculum was prepared by suspending a loop full of bacterial cultures into 10 mL of nutrient agar broth and was incubated at 37 °C for 24 h. About 60 µL of bacterial suspensions, adjusted to 10^6^–10^7^ CFU/mL were taken and poured into Petri plates containing 10 mL sterilized nutrient agar medium. Bacterial suspensions were spread to get a uniform lawn culture [63].

#### 3.6.2. Antimicrobial Activity Assay

Antimicrobial activities of the *L. barbarum* and *L. chinense* extracts were evaluated by means of agar-well diffusion assay with some modifications [63]. Fifteen millilitres of the molten agar (45 °C) were poured into sterile Petri dishes (Ø 90 mm). Cell suspensions were prepared and 100 µL was evenly spreader onto the surface of the agar plates of Mueller-Hinton agar (Oxoid, Basingstoke, UK). Once the plates had been aseptically dried, 6 mm wells were punched into the agar with a sterile Pasteur pipette. The different extracts (10 mg/mL) were dissolved in dimethylsulfoxide/water (1/9) and 80 µL were placed into the wells and the plates were incubated at 37 °C for 24 h. Gentamicin (10 g/wells) was used as positive control for bacteria. Antimicrobial activity was evaluated by measuring the diameter of circular inhibition zones around the well. Tests were performed in triplicate and values are the averages of three replicates.

#### 3.6.3. Minimum Inhibitory Concentration

Based on the previous screening the minimum inhibitory concentration (MIC) of both *L. barbarum* and *L. chinense* extracts were analyzed through the agar-well diffusion method. A bacterial suspension (10^5^–10^6^ CFU/mL) of each tested microorganism was spread on the nutrient agar plate. The wells (6 mm diameter) were cut from agar, and 60 µL of *L. barbarum* and *L. chinense* extracts dissolved in DMSO at different concentrations (10, 20, 25, 75, 100 µg/mL) were delivered into them. The plates were incubated at 37 °C for 24 h under aerobic conditions that followed by the measurement of the diameter of the inhibition zone expressed in millimeter. MIC was taken from the concentration of the lowest dosed well visually showing no growth after 24 h [1,58,59].

### 3.7. Statistical Analysis

A statistical approach was designed and the experimental data were evaluated using one-way analysis of variance (ANOVA), with *p* < 0.05 as threshold for statistical significance. The statistical results confirm the hypothesis that the differences between the results are either not significant (*p* > 0.05), significant (0.001 < *p* < 0.05) or highly significant (*p* < 0.001). The average of multiple measurements (triplicates or more) was listed in the tables together with the standard deviations. Statistical analysis was performed using Excel software package.

## 4. Conclusions

We analyzed the polyphenols from the leaves of two *Lycium* species known as important functional foods, *L. barbarum* and *L. chinense*, and we have completed the literature data with new information regarding the polyphenolic compounds from *Lycium* species. Phytochemical investigations suggest these species as important sources of flavonoids and chlorogenic acid. The results of the antioxidant assays showed a good correlation between methods as well as with the content of total polyphenols with a relevant antioxidant activity in both extracts, *L. chinense* extract exhibiting a higher antioxidant activity than *L. barbarum*. Results obtained in the antimicrobial tests relieved that *L. chinense* extract was found to be more active than *L. barbarum* against both Gram-positive and Gram-negative bacterial strains. The best antibacterial activity was shown by *L. chinense* extract against *Bacillus subtilis*. Regarding the MIC results, *L. chinense* and *L. barbarum* extracts exhibited good antimicrobial activity against most of the tested microorganisms. Summarizing the results of the present study we can conclude that both *L. barbarum* and *L. chinense* leaves are valuable sources of flavonoids with important antioxidant and antimicrobial activities.
